# Tissue and nitrogen-linked expression profiles of ammonium and nitrate transporters in maize

**DOI:** 10.1186/s12870-019-1768-0

**Published:** 2019-05-20

**Authors:** Julie Dechorgnat, Karen L. Francis, Kanwarpal S. Dhugga, J. Antony Rafalski, Stephen D. Tyerman, Brent N. Kaiser

**Affiliations:** 1 University of Adelaide, School of Agriculture Food and Wine, 2B Hartley Grove, Urrbrae, SA 5064 Australia; 2 University of Sydney, School of Life and Environmental Sciences, 380 Werombi Road, Brownlow Hill, NSW 2570 Australia; 3 Genetic Discovery Group, DuPont Pioneer, Johnston, IA 50131-1004 USA; 4 Present Address: Genetic Resources Group, International Center for Maize and Wheat Improvement (CIMMYT), El Batan, 56237 Texcoco, Mexico; 5 Genetic Discovery Group, DuPont Crop Genetics Research, DuPont Experimental Station, Building E353, Wilmington, DE 198803 USA

**Keywords:** Maize (*Zea mays*), Transporters, Nitrate, Ammonium, Gene expression

## Abstract

**Background:**

In order to grow, plants rely on soil nutrients which can vary both spatially and temporally depending on the environment, the soil type or the microbial activity. An essential nutrient is nitrogen, which is mainly accessible as nitrate and ammonium. Many studies have investigated transport genes for these ions in *Arabidopsis thaliana* and recently in crop species, including Maize, Rice and Barley. However, in most crop species, an understanding of the participants in nitrate and ammonium transport across the soil plant continuum remains undefined.

**Results:**

We have mapped a non-exhaustive set of putative nitrate and ammonium transporters in maize. The selected transporters were defined based on previous studies comparing nitrate transport pathways conserved between Arabidopsis and *Zea mays* (Plett D et. al, PLOS ONE 5:e15289, 2010). We also selected genes from published studies (Gu R et. al, Plant and Cell Physiology, 54:1515-1524, 2013, Garnett T et. al, New Phytol 198:82-94, 2013, Garnett T et. al, Frontiers in Plant Sci 6, 2015, Dechorgnat J et. al, Front Plant Sci 9:531, 2018). To analyse these genes, the plants were grown in a semi-hydroponic system to carefully control nitrogen delivery and then harvested at both vegetative and reproductive stages. The expression patterns of 26 putative nitrogen transporters were then tested. Six putative genes were found not expressed in our conditions. Transcripts of 20 other genes were detected at both the vegetative and reproductive stages of maize development. We observed the expression of nitrogen transporters in all organs tested: roots, young leaves, old leaves, silks, cobs, tassels and husk leaves. We also followed the gene expression response to nitrogen starvation and resupply and uncovered mainly three expression patterns: (i) genes unresponsiveness to nitrogen supply; (ii) genes showing an increase of expression after nitrogen starvation; (iii) genes showing a decrease of expression after nitrogen starvation.

**Conclusions:**

These data allowed the mapping of putative nitrogen transporters in maize at both the vegetative and reproductive stages of development. No growth-dependent expression was seen in our conditions. We found that nitrogen transporter genes were expressed in all the organs tested and in many cases were regulated by the availability of nitrogen supplied to the plant. The gene expression patterns in relation to organ specificity and nitrogen availability denote a speciality of nitrate and ammonium transporter genes and their probable function depending on the plant organ and the environment.

**Electronic supplementary material:**

The online version of this article (10.1186/s12870-019-1768-0) contains supplementary material, which is available to authorized users.

## Background

Nitrogen is an essential nutrient required for plant growth. It is a primary constituent of nucleic acids, amino acids and proteins. Although 78% of the atmospheric air is made of N_2_, only legumes are able to convert atmospheric N_2_ to plant available forms of nitrogen via a symbiotic biological process involving *Rhizobium* bacteria resident in plant roots [1]. Plants not capable of fixing N_2_ absorb it through their roots mainly in the form of the inorganic ions, nitrate (NO_3_^−^) in aerobic soils and ammonium (NH_4_^+^) in acidic soils and wetlands. Once absorbed, NO_3_^−^ and NH_4_^+^ undergo a complex process of assimilation, transformation and mobilization within the plant [2–5]. In agricultural soils, cereal crops often fail to access half of the nitrogen fertilisers applied by farmers [6–8]. The excessive and inefficient use of nitrogen fertilisers, coupled with the low absorption capacity of crops, results in leaching of NO_3_^−^ after rainfall or irrigation events [9]. This causes contamination of ground water and in many cases excessive algal growth in rivers and deltas leading to eutrophication and subsequently death of aquatic life. Reactive nitrogen is also lost through atmospheric release of gaseous forms of nitrogen including nitrous oxide, a potent greenhouse gas. A better understanding of nitrogen uptake and distribution within the plant is important for genetically engineering improvements in nitrogen use efficiency and nitrogen utilisation for yield and quality in crop species.

The uptake of both NO_3_^−^ and NH_4_^+^ involves two physiological mechanisms [10, 11]. When nitrogen concentrations are low (< 250 μM), a high affinity transport system (HATS) is observed. This low-capacity HATS is under the genetic control of the *NRT2* (Nitrate Transporter 2) and *AMT1* (Ammonium Transporter 1) families for NO_3_^−^ and NH_4_^+^, respectively [4, 12]. Conversely, when nitrogen concentrations are high (> 250 μM), a low-affinity transport system (LATS) becomes active. The NO_3_^−^ LATS involves the *NPF* (NRT1/PTR Family) gene family [4, 13]. Although no transporter for the NH_4_^+^ LATS has been described yet, the recent identification of AMF1 (Ammonium Facilitator 1) proteins in soybean and yeast are promising candidates [14].

Four *ZmNRT2* genes have been identified in the maize genome: *ZmNRT2.1*, *ZmNRT2.2*, *ZmNRT2.3* and *ZmNRT2.5* [15]. Only two members of the family have been studied, *ZmNRT2.1* and *ZmNRT2.2* Both genes are closely related, sharing 98% homology in their amino acids sequence [16]. Both are NO_3_^−^ inducible genes in seedling roots [16–18]. In situ analysis in seedling roots revealed a specific localisation of *ZmNRT2.1* transcripts in the cortex whereas *ZmNRT2.2* transcripts could be found in the cortex, the stele and the incipient of root lateral primordia [16]. The expression of both genes has been detected in seedling shoots but at a much lower level than in the roots [16, 18]. NRT2.1 proteins have been shown to be part of a plasma membrane tetramer complex with NRT3.1 proteins (also known as NAR2.1). This complex forms a functional unit responsible for HATS influx in roots [19–21]. The maize genome contain two copies of the *NRT3.1* gene: *ZmNRT3.1A* and *ZmNRT3.1B* [15], neither have been characterised in maize.

Ten genes belonging to the NPF family have been discovered in maize [15]. Little is known about their expression, localisation or function. Only two members of the family have been recently characterised. ZmNPF6.4 is a low affinity NO_3_^−^ transporter with efflux activity that has been reported as a potential high-affinity chloride transporter [22]. The gene is expressed in both shoots and roots of maize seedlings independently of the nitrogen concentration in the environment. ZmNPF6.6, in contrast, was found to participate in the high-affinity NO_3_^−^ specific transport in a pH-dependent manner [22]. Transcripts of the corresponding gene were detected mainly in the roots of maize seedlings. When plants were grown in hydroponics, *ZmNPF6.6* expression was downregulated after 4 days of nitrogen starvation and upregulated specifically after NO_3_^−^ resupply [22].

The maize *AMT* family contains 8 members divided in four classes, *ZmAMT1* to *ZmAMT4* [23], but only three genes have been previously studied [24]. *ZmAMT1.1A, ZmAMT1.1B* and *ZmAMT1.3* belong to the sub-class 1 of the AMT family. *ZmAMT1.1B* is a low expressed gene in maize. *ZmAMT1.1A* and *ZmAMT1.3* are expressed in the rhizodermis of the apical root zone and act as high affinity NH_4_^+^ transporters with *K*m affinities of 48 and 33 μM, respectively [24]. NH_4_^+^ supply specifically up-regulates the expression of *ZmAMT1.1a* and *ZmAMT1.3* independently of the whole plant nitrogen status [24].

Although the low affinity transport of NH_4_^+^ was described in rice roots more than 20 years ago [25], no LATS transporter has been described yet in plants. Potential candidates are AMF1 (Ammonium Facilitator 1) proteins. These proteins were discovered in an heterologous expression system in yeast [14]. The ectopic expression of a soybean transcription factor, *GmbHLHm1*, in yeast activated the expression of *ScAMF1* which allowed a NH_4_^+^ transport-deficient strain of *Saccharomyces cerevisiae* to grow on an NH_4_^+^ enriched-medium. A phylogenetic analysis revealed the presence of *AMF1* genes in most plants [14]. In maize, the family is composed of two members, *ZmAMF1.1* and *ZmAMF1.2.*

In recent years, an increasing collection of informative transcriptional gene array data sets have been presented in public sites such as Maize GDB [26]. This data is useful for contextual understanding of putative gene expression profiles, particularly tissue profiles which support high levels of gene expression. However, in the context of understanding transcriptional networks linked to nitrogen utilisation, direct measurement of gene expression combined with targeted physiological sample preparation is often the preferred method supporting gene discovery. With this in mind, we set out to better understand the spatial distribution of nitrogen transporters in maize by creating a map of gene expression patterns for nitrogen transporters expressed in both vegetative and reproductive stages of maize development. A selection of these genes was then analysed for their response to continual nitrogen supply, starvation and resupply. The data indicates a preferential gene response of nitrate and ammonium transporters linked to nitrogen availability across multiple regions of the plant.

## Results

**Fig. 1:**
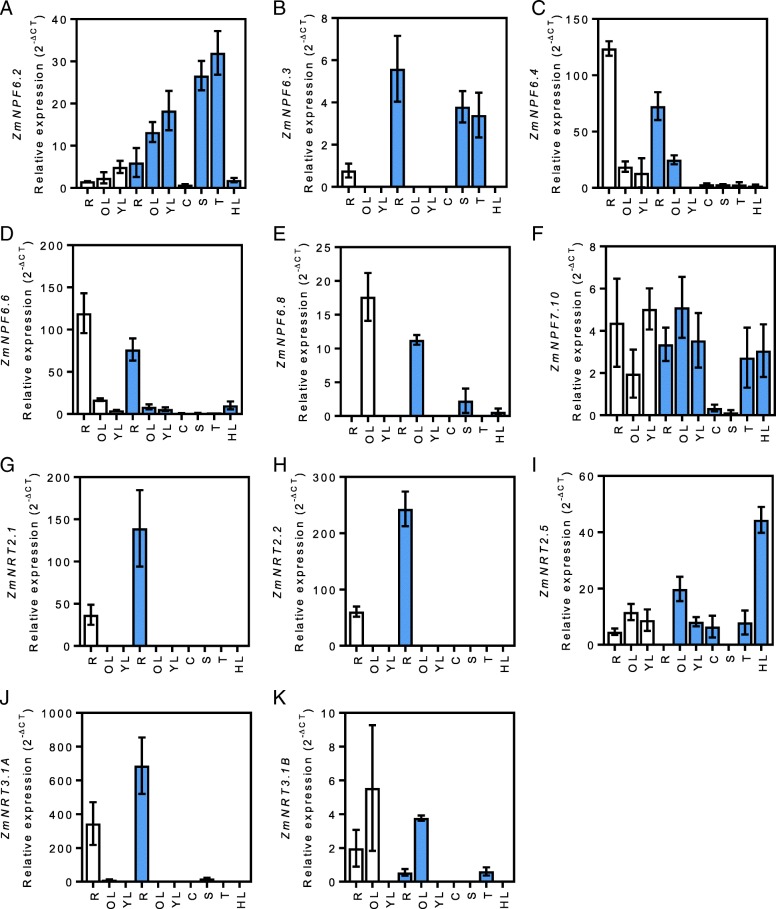
Nitrate transporter expression profiles. Relative expression (2^-∆CT^) of *ZmNPF6.2* (**a**), *ZmNPF6.3* (**b**), *ZmNPF6.4* (**c**), *ZmNPF6.6* (**d**), *ZmNPF6.8* (**e**), *ZmNPF7.10* (**f**), *ZmNRT2.1* (**g**), *ZmNRT2.2* (**h**), *ZmNRT2.5* (**i**), *ZmNRT3.1A* (**j**) and *ZmNRT3.1B* (**k**) in roots (r), old leaves (OL), young leaves (YL), cobs (c), silks (**s**), tassels (**t**) and husk leaves (HL) during V7 vegetative (white) and R1 reproductive (blue) development stages. Gene expression is calculated as relative expression to four control genes (*ZmUBQc*, *ZmSIN3*, *ZmCullin* and *ZmElF1*). Values are means (±SE) from 3 individual plants

**Fig. 2:**
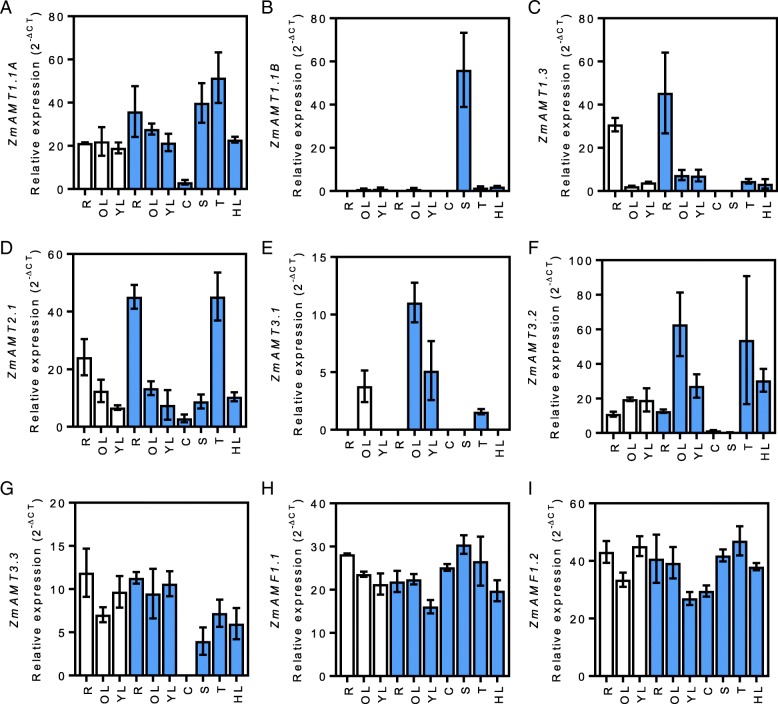
Ammonium transporter expression profiles. Relative expression (2^-∆CT^) of *ZmAMT1.1A* (**a**), *ZmAMT1.1B* (**b**), *ZmAMT1.3* (**c**), *ZmAMT2.1* (**d**), *ZmAMT3.1* (**e**), *ZmAMT3.2* (**f**), *ZmAMT3.3* (**g**), *ZmAMF1* (**h**) and *ZmAMF2* (**i**) in roots (R), old leaves (OL), young leaves (YL), cobs (C), silks (S), tassels (T) and husk leaves (HL) during V7 vegetative (white) and R1 reproductive (blue) development stages. Gene expression is calculated as relative expression to four control genes (*ZmUBQc*, *ZmSIN3*, *ZmCullin* and *ZmElF1*). Values are means (±SE) from 3 individual plants

**Fig. 3:**
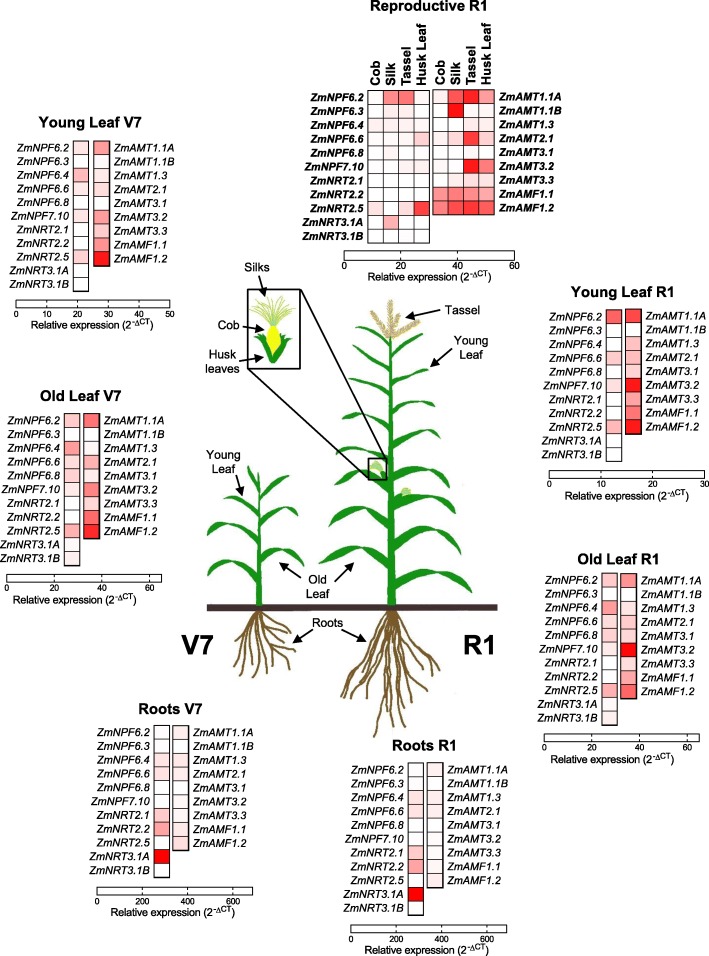
Heatmap (2^-∆CT^) representation of nitrate and ammonium transporter relative gene expression relative to control genes (*ZmUBQc*, *ZmSIN3*, *ZmCullin* and *ZmElF1*) (Data from Fig. 1) in maize plants at vegetative stage V7 and reproductive stage R1

In order to study the expression profiles of maize nitrogen transporter genes, plants were grown using a semi-hydroponic system in the glasshouse that enabled full-plant growth with the ability to access aerial and root samples. Plants were harvested at a vegetative (V7) and reproductive stage (R1) of growth from which RNA was extracted from a range of tissues that included roots, old leaves, young leaves, cobs, silks, tassels and husk leaves (Fig. 1). A set of sixteen nitrogen transport genes were identified (Additional file 1: Table S1) from previous maize studies examining nitrate and ammonium transport [24, 27–29] as well as genes extracted from the maize genome which have been identified to be closely related to characterised *Arabidopsis thaliana* transport genes [15]. Among the sixteen putative NO_3_^−^ transporter genes included in this study, five were found to be not expressed across the tissue samples examined. No transcripts were detected for *ZmNPF4.10*, *ZmNPF6.5*, *ZmNPF6.7, ZmNPF7.12* and *ZmNRT2.3* (data not shown). However, each of the remaining genes were expressed at both growth stages at varying levels within the tissues sampled. Although growth-dependent expression was generally absent, differences in expression levels occurred between the vegetative and reproductive tissue stages for most of the genes examined (Fig. 1). In parallel, the expression profile of genes linked to ammonium transport (AMT and AMF) were also explored (Fig. 2). In general, most *AMT* and *AMF* genes were found to be expressed across the tissues tested at V7 and R1 stages. Tissue specific expression in stems was identified for ZmAMT1.1B while root expression across both growth stages was present for *ZmAMT1.3* (Fig. 2B, C). We have summarised this data in colour indicative heat maps to help visualise the gene expression patterns of nitrate and ammonium transporters across tissue types and the two developmental stages, vegetative (V7) and reproductive (R1). Gene expression is presented relative to that of tested control genes (Fig. 3). The data clearly illustrates significant changes in expression of ammonium (*AMT* and *AMF*) genes in reproductive tissues and as leaves transition from young to older leaves.

**Fig. 4:**
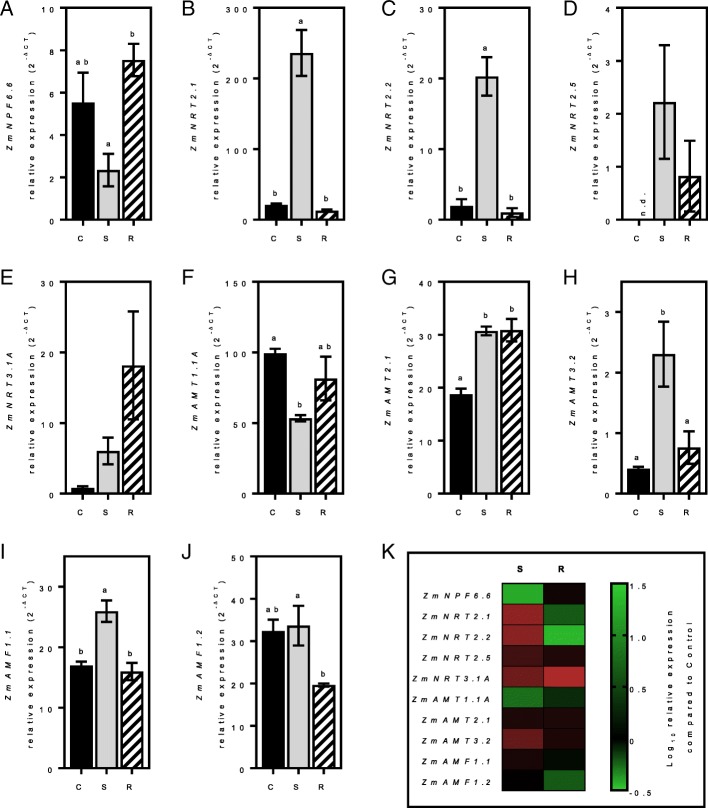
Root gene expression in response to nitrogen. Relative expression (2^-∆CT^) of *ZmNPF6.6* (**a**), *ZmNRT2.1* (**b**), *ZmNRT2.2* (**c**), *ZmNRT2.5* (**d**), *ZmNRT3.1A* (**e**), *ZmAMT1.1A* (**f**), *ZmAMT2.1* (**g**), *ZmAMT3.2* (**h**), *ZmAMF1.1* (**i**) and *ZmAMF1.2* (**j**) in control (C), starved (S) or resupplied (R) roots. Summary of gene expression between starved and resupplied root tissues (**k**). Gene expression is calculated as relative expression to four control genes (*ZmUBQc*, *ZmSIN3*, *ZmCullin* and *ZmElF1*). Values are means (±SE) from 3 individual plants. The significance of differences between the values was assessed by one-way ANOVA test

**Fig. 5:**
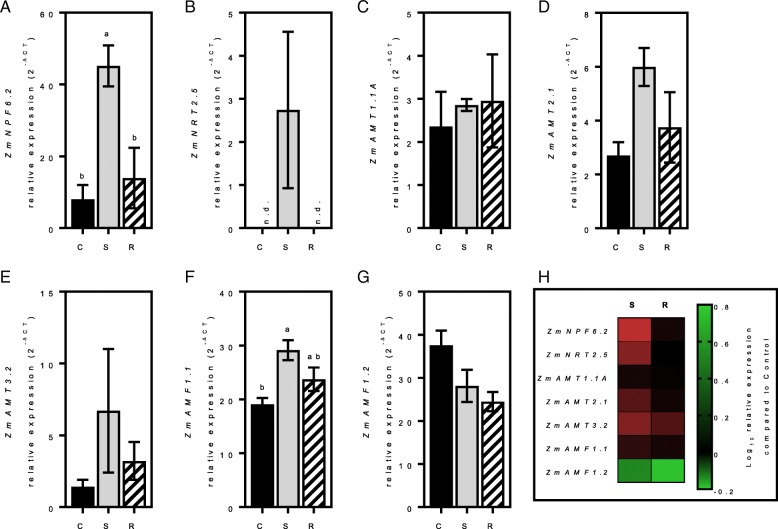
Shoot gene expression in response to nitrogen. Relative expression (2^-∆CT^) of *ZmNPF6.2* (**a**), *ZmNRT2.5* (**b**), *ZmAMT1.1A* (**c**), *ZmAMT2.1* (**d**), *ZmAMT3.2* (**e**), *ZmAMF1.1* (**f**) and *ZmAMF1.2* (**g**) in control (C), starved (S) or resupplied (R) shoots. Summary of changes in gene expression between starved and resupplied shoots (**h**). Gene expression is calculated as relative expression to four control genes (*ZmUBQc*, *ZmSIN3*, *ZmCullin* and *ZmElF1*). Values are means (±SE) from 3 individual plants. The significance of differences between the values was assessed by one-way ANOVA test

In order to study gene expression response to nitrogen in roots and shoots (Figs. 4 and 5, respectively), plants were grown in hydroponics on a controlled nitrogen diet containing a nitrogen supplied medium (C) for 17 days. The plants were then starved of nitrogen for four days (S) before a 24 h-nitrogen resupply (R). The procedure for these experiments followed existing protocols we have used successfully to examine the impact of nitrogen supply, starvation and re-supply on gene expression and plant growth in maize and other plant systems [27–31]. Genes presenting low or no expression in young plants were omitted in this analysis (i.e. *ZmNPF4.10*, *ZmNPF6.3*, *ZmNPF6.5*, *ZmNPF6.7*, *ZmNPF6.8*, *ZmNPF7.10*, *ZmNPF7.12*, *ZmNRT2.3*, *ZmNRT3.1B*, *ZmAMT1.1B*, *ZmAMT3.1* and *ZmAMT4*). Although expressed, *ZmNPF6.4*, *ZmAMT1.3* and *ZmAMT3.3* did not respond to the nitrogen starvation (data not shown). The most nitrogen responsive genes were identified as *ZmNRT2.1* and *ZmNRT2.2,* where expression increased more than 10-fold in roots after starvation (Fig. 4b, c, k). However, 24 h after nitrogen resupply, their expression returned to control levels. *ZmNRT2.1* and *ZmNRT2.2* are described as high-affinity NO_3_^−^ transporters, most active when the nitrogen concentrations provided to roots are low. In the shoots, only *ZmNPF6.2*, *ZmNRT2.5* and *ZmAMF1.1* responded significantly to a period of nitrogen starvation (Fig. 5). Both *ZmNPF6.2 ZmNRT2.5* expression decreased after nitrogen was resupplied to the plants.

## Discussion

### Nitrate transporters

The tissue and developmental expression patterns of maize nitrogen transport genes requires defining to help understand the underlying mechanisms that influence nitrogen uptake and redistribution in maize plants, both of which are key traits in improving nitrogen utilisation of cereal crops. In the context of nitrate transporters, *ZmNPF7.10* was the only gene to have a ubiquitous expression pattern independent of the age of the plant; however, its transcript levels were found to be quite low (Fig. 1f). Two members of the NPF7 family (*AtNPF7.3*, *AtNPF7.2*) have been previously described in *A. thaliana*. *AtNPF7.3*/*AtNRT1.5* encodes a low-affinity NO_3_^−^ transporter located in the root pericycle cells near the xylem and is responsible for xylem loading of NO_3_^−^ [32]. AtNPF7.3/AtNRT1.5 is also linked to the tolerance to cadmium, drought and salt stress as knock-out mutant plants displayed higher resistance to these abiotic stresses [33]. *AtNPF7.2*/*AtNRT1.8* also encodes a low-affinity NO_3_^−^ transporter [34] expressed within xylem parenchyma cells. AtNPF7.2/AtNRT1.8 is involved in the efflux of NO_3_^−^ from xylem vessels. Together, AtNPF7.3/AtNRT1.5 and AtNPF7.2/AtNRT1.8 are believed to work in concert with each other to load and unload NO_3_^−^ from the xylem, respectively. The maize *NPF7* family contains a total of 12 members [13] but only two members were analysed in our study. *ZmNPF7.12* was not expressed (data not shown) and *ZmNPF7.10* was found to be ubiquitously expressed across tissues and developmental stages (Fig. 1f). Given the similar orthology between the *A. thaliana* and the maize genes, the function of *ZmNPF7* genes in xylem loading/unloading is likely. A more detailed analysis of the *NPF7* family in maize would help better understand the control of NO_3_^−^ loading in the xylem.

The putative HATS transporter genes *ZmNRT2.1*, *ZmNRT2.2* and *ZmNRT3.1A* displayed similar root-specific expression profiles (Fig. 1 g, h and j respectively). Moreover, their root-specific expression was higher at R1 than at the V7 stage, reaching 139-, 243- and 687- fold levels of the control genes, respectively. These data confirmed previous results on the dominant localisation of *ZmNRT2.1* and *ZmNRT2.2* transcripts in maize roots [16, 18, 27]. The co-localised expression of *ZmNRT3.1A*, *ZmNRT2.1* and *ZmNRT2.2* tend to confirm the function of ZmNRT3 in maize as described by Lupini et al., 2016 [35]. The authors showed a functional interaction of ZmNRT2.1 with ZmNRT3.1A in regulating NO_3_^−^ uptake along the root axis of maize. The two-component NO_3_^−^ uptake system of NRT2-NRT3 has been demonstrated to be also present in other plant species such as *A. thaliana* [21, 36], barley [20] or rice [19]. We followed the expression profile of a second NRT3 gene, *ZmNRT3.1B*, and detected a higher expression in old leaves than in roots (Fig. 1k). However, *ZmNRT3.1B* expression is negligible compared to its homologue *ZmNRT3.1A*, which was expressed at a 100-fold lower level (Fig. 1 j and 1 k).

*ZmNRT2.5*, another putative HATS gene, was found expressed in all organs at low levels at the V7 stage (Fig. 1i). At the reproductive stage, no transcript could be detected in roots or silks. Although at R1, the gene was expressed in the leaves, cobs and tassels and its expression was significantly higher in the husk leaves (Fig. 1i). *ZmNRT2.5* was the only putative NO_3_^−^ transporter found expressed at high levels in the husk leaves. As husk leaves play a central role in the distribution of nitrogen during the grain filling period [37], the role of *ZmNRT2.5* in this process needs to be examined further.

The recently characterised genes *ZmNPF6.4*, coding a low-affinity NO_3_^−^ transporter [22], and *ZmNPF6.6*, coding a high affinity NO_3_^−^ transporter [22], displayed similar expression patterns (Fig. 1c and d respectively). Both genes were mainly expressed in the roots, although transcripts could be detected in other organs but at a significantly lower level. Both gene expression patterns were higher at the vegetative stage than the reproductive stage. We expect this anomaly was due to the reduced nitrogen uptake capacity of maize after flowering [27]. Indeed, post-silking only 35–55% of the grain nitrogen originates from nitrogen uptake, the rest provided from the pre-existing nitrogen stored before silking in leaves and stems [6, 38]. The root expression pattern of the two genes is in line with the results found previously for *ZmNPF6.4* and *ZmNPF6.6*, two of the main NO_3_^−^ root transporters [22, 29]. 

*ZmNPF6.8* was the only gene found with a specific expression pattern in old leaves for both vegetative and reproductive stages (Fig. 1e). In senescing leaves, programmed degradation of leaf proteins are an important source of remobilised nitrogen used to supplement growing organs, including grains or newly formed leaves [3, 38, 39]. The fact that *ZmNPF6.8* encodes a putative NO_3_^−^ transporter expressed in source organs makes it an important target gene to further explore leaf nitrogen remobilisation. Another avenue where ZmNPF6.8 may play an important role is in the transport of polyamines, a class of low molecular weight aliphatic polycations. In *A. thaliana*, the mutant line *sper3–3* shows an increased tolerance to toxic levels of polyamines [40]. The corresponding gene, *AtNRT1.3/AtNPF6.4*, a close orthologue to *ZmNPF6.8*, was found to be expressed in leaves, stems and flowers. Tong et al., 2016 [40] concluded that the transport or metabolism of polyamines is associated with the NO_3_^−^ transport activities in the parenchymal tissues of *A. thaliana* shoots.

*ZmNPF6.2* transcripts were detected mainly in leaves and gamete-producing organs (silk and tassel) (Fig. 1a). Its leaf expression was higher at R1 stage compared to V7. *ZmNPF6.2* is an orthologue of *AtNPF6.2/AtNRT1.4* [13, 15]. In *A. thaliana*, *AtNPF6.2/AtNRT1.4* encodes a NO_3_^−^ LATS transporter expressed in the petiole and the adjacent part of the midrib of the leaf [41]. This low-affinity NO_3_^−^ transporter may be involved in the regulation of leaf NO_3_^−^ homeostasis. Given the homology and expression pattern similarities between *AtNPF6.2* and *ZmNPF6.2*, it is possible that this transporter carries out the same function in both the plant species. The *ZmNPF6.2* homologue, *ZmNPF6.3* [15], was also expressed in the silks and tassels as well as in the roots but its expression was around 10-fold lower than *ZmNPF6.2* (Fig. 1b and a respectively).

### Ammonium transporters

The two putative NH_4_^+^ LATS transporter genes were constitutively expressed in our experiments. *ZmAMF1.1* and *ZmAMF1.2* expression was similar in all the organs independent of the growth stage (Fig. 2h and i, respectively). Moreover, both the genes had comparable levels of transcripts. The ubiquitous expression of *AMF1* genes in maize was surprising. Contrary to the nitrate transporters *ZmNPF6.6* and *ZmNPF6.8* that were specifically expressed in the roots and old leaves, respectively, (Fig. 1d and e), *ZmAMF1.1* and *ZmAMF1.2* seem to be present in every organ tested (Fig. 2h and i). The function of these two genes and their respective protein activities require further investigation.

Three genes of the *AMT1* family were found expressed in our experiment. *ZmAMT1.1A* was constitutively expressed at V7. Transcripts were also found at R1 and in all organs except the cobs where their levels were minor (Fig. 2a). Our results are in accordance with those previously described by Gu et al. [24] where they detected ubiquitous *ZmAMT1.1A* expression at the seedling and silking stages and 15 days post pollination [24]. *ZmAMT1.1A* expression patterns is conserved in other plant species, including rice (*OsAMT1.1)* and sorghum (*SbAMT1.1*) [23, 42]. Contrary to *ZmAMT1.1A*, its closest homologue, *ZmAMT1.1B* showed specific expression only in the silks (Fig. 2b). This particular pattern had already been seen by Gu et al. (2013), which showed an enhanced expression of *ZmAMT1.1B* in the immature ear at the silking stage [24]. A third member of the AMT1 family, *ZmAMT1.3*, was found specifically expressed in the roots independently of the growth stage of the plant (Fig. 2c). A similar pattern has been described at the seedling stage [24] . However, in the reproductive stage, *ZmAMT1.3* was also expressed in the leaves, while the data for root expression is still to be determined [24]. Our results show a high level of expression in root tissues that may indicate a role of ZmAMT1.3 in the root NH_4_^+^uptake.

The only known member of the AMT2 family in maize, *ZmAMT2.1,* was found expressed in all organs with some specificity to roots and tassels (Fig. 2d). Interestingly, Koegel et al. [23] indicated a similar expression pattern of the *ZmAMT2.1* orthologue in sorghum. Indeed, the authors showed that in sorghum, *SbAMT2.1* was expressed in all organs studied with a higher expression in roots and stamens. This analogous profile shows a conservation of expression patterns between species. Functional analysis is required to assess the conservation of function between ZmAMT2.1 and SbAMT2.1.

Transcripts of *ZmAMT3.2* were detected in all organs but were higher in the older leaves (Fig. 2f). High expression could also be seen in the tassels although the data was variable and not conclusive. Its homologue, *ZmAMT3.3*, was also expressed in all organs except in the cobs (Fig. 2g). The broad expression patterns of *ZmAMT3.2* and *ZmAMT3.3* are similar to their close orthologues in sorghum [23]. Transcripts of *SbAMT3.2* and *SbAMT3.3* were detected in roots, stems, shoots and pistils of field grown plants [23]. *SbAMT3.3* was also expressed in the stamens of sorghum. However, the function of the transporters and their involvement in the NH_4_^+^ transport has yet to be demonstrated.

Transcripts of the last member of the *AMT3* family, *ZmAMT3.1*, were detected only in the OL at V7 and in the leaves and tassels at R1. This contrasts with the previous finding in sorghum that showed *SbAMT3.1* to be expressed mainly in the roots [23]. A detailed functional analysis of these two genes is required to resolve the dissimilarity in expression patterns between maize and sorghum. The only known member of the *AMT4* family in maize, *ZmAMT4*, was the only AMT gene found not to be expressed in our samples (data not shown).

To provide a visual summary of indicative gene expression at the vegetative (V7) and reproductive stages (R1), gene expression relative to controls have been presented as colour indicative heat maps (Fig. 3). There is a clear definition in the expression of both nitrate and ammonium transport genes across the tissues and the two development phases of the plants. In the context of nitrogen transporter activity in reproductive tissues, ammonium transport (*AMT1*, *AMT2* and *AMF1*) systems are clearly induced with expected roles in nitrogen redistribution in these important tissues. Activity of nitrate transport systems in flowering tissues (R1) are noticeably less than those of ammonium.

### Response to nitrogen

Following starvation, we expect the expression of the HATS encoding genes to increase to compensate for the reduction of external nitrogen [43]. A similar pattern has been previously described in *A. thaliana* [44, 45]. In hydroponically grown plants, *AtNRT2.1* was demonstrated to be expressed rapidly and strongly after nitrogen starvation [45] peaking 24 h after the start of the experiment. The other NO_3_^−^ HATS gene was found responsive in both roots and shoots. No expression of *ZmNRT2.5* could be detected in our control conditions probably because of the younger age of the plants used in the starvation experiment (Fig. 4D and 5B). However, *ZmNRT2.5* expression increased after starvation in both organs before decreasing after nitrogen resupply (Fig. 4D and 5B). In *A. thaliana*, the orthologous gene, *AtNRT2.5*, was also found to be induced after nitrogen starvation [45]. The authors demonstrated that gene expression increased during the starvation period. These results highlight a conserved expression pattern of *NRT2* genes between species. A deeper analysis of the *NRT2* genes and their protein activities will be required to confirm if functional conservation exists between species.

*ZmNRT3.1A* expression followed the pattern of its putative partners, *ZmNRT2.1* and *ZmNRT2.2*, as it increased during starvation in the roots (Fig. 4e, k). However, *ZmNRT3.1A* expression remained elevated after resupply. A longer period of resupply may be needed to detect a decrease in its expression. A comparable pattern of expression between *NRT2* and *NRT3* genes has previously been demonstrated in *A. thaliana*. Orsel et al. (2006) indicated that *AtNAR2.1/AtNRT3.1* expression increased after 24 h of NO_3_^−^ starvation in a similar fashion to *AtNRT2.1* [46]. The authors concluded that, since both proteins are required for functional HATS activity, their expression should be closely coordinated with the expression of both At*NRT2.1* and At*NAR2.1/AtNRT3.1* components. A corresponding protein association of ZmNRT2.1 and ZmNRT3.1A was recently demonstrated in maize roots [47]. Our results are in accordance with these previous findings.

Although not significant, the expression of *ZmNPF6.6* decreased after nitrogen starvation (~ 58%) but then returned to base level after nitrogen resupply (Fig. 4a, k). These results are in agreement with the recently published work by Wen et al. [22] where the authors described a decrease of *ZmNPF6.6* expression after root starvation. This phenotype was reversible with the resupply of NO_3_^−^. Still, it is unknown whether the modulation of *ZmNPF6.6* expression translate into a decrease of the transporter activity.

The only shoot specific putative NO_3_^−^ transporter found responsive to changes in nitrogen supply was *ZmNPF6.2*. Transcripts increased (5.6-fold) after starvation and then reverted to initial levels after nitrogen resupply (Fig. 5a, h). Chiu et al. (2004) demonstrated the role of AtNPF6.2/AtNRT1.4 in NO_3_^−^ storage of the petiole. The authors showed that, in the *Atnrt1.4* mutant, the accumulation of NO_3_^−^ in the petiole was reduced by half compared to the wild-type levels [41]. The petiole NO_3_^−^ content is commonly used as a rapid diagnostic test of the plant nitrogen status and an indicator of yield response in many crops like capsicum, cotton or potatoes [48–52]. Hence, *AtNPF6.2/AtNRT1.4* might be an important marker of plant nitrogen status. In our experiments, *ZmNPF6.2* expression responded to the availability of nitrogen to the plant which would support an involvement in the regulation of petiole NO_3_^−^ content as seen in *A. thaliana*. The subcellular localisation of NPF6.2/NRT1.4 in both *A. thaliana* and maize needs to be validated.

*ZmAMT1.1A* transcripts were detected in most organs (Fig. 2a) however their individual responses were variable. In the roots, *ZmAMT1.1A* expression decreased by 46% after nitrogen starvation and slowly rose after resupply (Fig. 4f, k). On the other hand, the gene was found unresponsive to nitrogen in the shoots (Fig. 5c, h). This lack of response in the shoot might be due to a specificity of the gene to the roots. Previous studies already described the diminution by nearly half of *ZmAMT1.1A* transcripts following a nitrogen starvation treatment [24]. Our data confirmed *ZmAMT1.1A* expression is nitrogen dependent in roots.

Although expressed in different organs, *ZmAMT2.1* and *ZmAMT3.2* presented a similar response to nitrogen in maize seedlings. In both roots and shoots, *ZmAMT2.1* showed an upregulation of expression after nitrogen starvation (Fig. 4G, K and 5D, H). This expression returned to control levels after resupply in the shoots whereas in the roots, even after 24 h of nitrogen, the levels of *ZmAMT2.1* were still similar to the starvation condition. A longer period of resupply might be necessary to see downregulation of *ZmAMT2.1* in the roots. Only one gene of the *AMT3* family was found to be responsive to nitrogen in both shoot and root. *ZmAMT3.2* expression increased by nearly 5-fold in both roots and shoots after nitrogen starvation, although it was not significant in the shoots (Fig. 4H, K and 5E, H). After resupply, the transcript levels returned to base levels. The analogous nitrogen response of *ZmAMT2.1* and *ZmAMT3.2* was opposite to the pattern of *ZmAMT1.1A* where root expression decreased after nitrogen starvation (Fig. 4f, k). These results highlight different mechanisms in maize in response to an abiotic stress. However, a confirmation of ZmAMT2.1 and ZmAMT3.2 protein involvement in NH_4_^+^ uptake is required.

Although expressed similarly in all organs, *ZmAMF1.1* and *ZmAMF1.2* presented different responses to nitrogen. In both roots and shoots, *ZmAMF1.1* expression increased by 1.5-fold after nitrogen starvation and decreased after resupply (Fig. 4I, K and 5F, H). *ZmAMF1.2*, on the other hand, did not present any significant response to nitrogen starvation but the transcript levels decreased after resupply.

## Conclusion

The analysis of nitrogen transporter genes in maize in different organs show that a given transporter can be specifically expressed in a tissue and a developmental stage as observed with the expression of *ZmNRT2.1* in the roots or *ZmAMT1.1B* in the silks (Figs. 1 and 5). Transcript abundances clearly overlap between genes, such as *ZmAMF1.1* and *ZmNRT7.10*. In most cases, the cellular localisation of each individual gene still needs to be tested and defined. Our results demonstrated that nitrogen transport genes are expressed in most of the tissues tested, but have divergent nitrogen regulation profiles as illustrated in Fig. 5. This non-exhaustive analysis has shown that both NO_3_^−^ and NH_4_^+^ transporter genes in maize participate in spatially separated expression patterns and transcriptional regulatory controls that allow the plant to respond to varying nitrogen conditions in the environment and across the development phases of plant growth. The results taken together will help build the foundation for future studies investigating NO_3_^−^ and NH_4_^+^ nutrition in maize and other crop species and to deliver outcomes that enable improved nitrogen utilisation across the growth cycle.

## Methods

### Plant material and growth conditions

Maize (*Zea mays L*., genotype B73) seeds were first imbibed 24 h in bubbling reverse osmosis water before sown individually in pots (diameter of 25 cm) filled with diatomaceous earth rocks (Maidenwell Diatomite Pty. Ltd., Australia). The plants were watered via a drip irrigation system and grown in a heated glasshouse. After one week on reverse osmosis water, the plants were supplied with a solution of 7 mM nitrogen containing 0.5 mM MgSO_4_, 0.5 mM KH_2_PO_4_, 0.05 mM KCl, 0.5 mM K_2_SO_4_, 2.5 mM KNO_3_, 0.75 mM CaCl_2_, 1.25 mM Ca(NO_3_)_2_, 1 mM NH_4_NO_3_, 0.1 mM Fe-EDTA, 0.1 mM Fe-EDDHA, 25 μM H_3_BO_3_, 2 μM MnSO_4_, 2 μM ZnSO_4_, 0.5 μM CuSO_4_ and 0.5 μM Na_2_MoO_4_. The nutrient solution was changed weekly to maintain nutrient levels and solution pH around 5.9.

The plants were harvested at a vegetative stage (V7) and a reproductive stage (R1) (Fig. 5). Roots were separated from the shoots and a representative sample was chosen. The second leaf and a young fully extended leaf were harvested and labelled OL (old leaf) and YL (young leaf) respectively. At the R1 stage, cobs, silks, tassels and husk leaves were harvested separately. All samples were snap-frozen in liquid nitrogen before analysis.

### Starvation experiment

Seeds were imbibed in bubbling reverse osmotic water for 4 h before sown individually onto a supportive mesh within a PVC seedling tube which contained moist diatomaceous earth rocks (Maidenwell Diatomite Pty. Ltd., Australia). 4 day-old seedlings were transferred to a 700 L ebb-and-flow hydroponic system that allows for continual 15 min fill/drain cycles. The seedling tubes were placed within larger tubes (300 mm × 50 mm), which kept the roots of adjacent plants separate, but allowed for free access of the roots to nutrient solution. The hydroponic system was situated in a controlled environment room with a day/night cycle of 12 h/12 h, temperatures of 28 °C and 21 °C respectively, and a light intensity of 300 μmol.m^− 2^.s^− 1^ at canopy level. The plants were supplied with nutrient solution containing 2.5 mM NH_4_NO_3_, 0.5 mM MgSO_4_, 0.5 mM KH_2_PO_4_, 1.05 mM KCl, 1.25 mM K_2_SO_4_, 0.25 mM CaCl_2_, 1.75 mM CaSO_4_, 0.1 mM Fe-EDTA, 0.1 mM Fe-EDDHA, 25 μM H_3_BO_3_, 2 μM MnSO_4_, 2 μM ZnSO_4_, 0.5 μM CuSO_4_ and 0.5 μM Na_2_MoO_4_. The nutrient solution was changed weekly to maintain nutrient levels and solution pH around 5.9.

After 17 days in the ebb-and-flow system, the plants assigned to the starvation treatment were washed with reverse osmosis water and exposed to the nutrient solution without nitrogen. Then 4 days later, selected starved plants were resupplied for 24 h with nutrient solution containing 2.5 mM NH_4_NO_3_. Twenty-six-day-old control, starved and resupplied plants were harvested at midday on the same day (Fig. 5). Roots were separated from shoots and samples were snap-frozen in liquid nitrogen.

### Gene expression analysis

Total RNA was isolated using the TRIzol Reagent (Life Technologies, Carlsbad, CA, USA) according to manufacturer’s protocol. RNA concentrations were estimated using a NanoDrop ND-1000 Spectrophotometer (Thermo Scientific, Waltham, MA, USA). cDNA was generated using 1 μg of total RNA using SuperScript III Reverse Transcriptase kit (Life Technologies, Carlsbad, CA, USA). cDNA was mixed with TaqMan OpenArray® Real-Time PCR Master Mix (Life Technologies™, Carlsbad, CA, USA). All samples were run on a QuantStudio 12 K Flex Real-Time PCR (RT-PCR) System (Life Technologies, Carlsbad, CA, USA) using 48-well plates TaqMan® OpenArray® RT PCR Inventoried Format 112 (Life Technologies™, Carlsbad, CA, USA). Four genes, ubiquitin-conjugating enzyme (*ZmUBQc*), SIN3 component, histone deacetylase complex (*ZmSIN3*), Cullin (*ZmCullin*) and Elongation factor 1-alpha (*ZmElF1*), were chosen as housekeeping genes to normalise gene expression data. Symbol numbers and genome gene ID are described in Additional file 1: Table S1.

### Statistical analysis

Values are given as mean ± standard error. Gene expression data in response to nitrogen were analysed by one-way analysis of variance (ANOVA). Differences in treatment levels were further evaluated for significance with Tukey post-hoc comparisons and a level of *P* < 0.05 was considered significant. These results are presented in the Additional file 1: Tables S2 and S3 for roots and shoots, respectively.

## Additional file

Additional file 1: **Table S1.** List of genes studied. **Table S2.** One-way ANOVA results of root gene expression between control (C), starved (S) and resupplied (R) plants. Bold text denotes *p* < 0.05. **Table S3.** One-way ANOVA results of shoot gene expression between control (C), starved (S) and resupplied (R) plants. Bold text denotes *p* < 0.05. (DOCX 118 kb)
