# Supplementary material for: Tissue and nitrogen-linked expression profiles of ammonium and nitrate transporters in maize
Source: BMC Plant Biol. 2019 May 20;19:206. doi: 10.1186/s12870-019-1768-0 (PMC6528335; doi:10.1186/s12870-019-1768-0)

**Supplemental Tables**

*Table S1*: List of gene studied

| Name | Gene ID |  | Name | Gene ID |  | Name | Gene ID |
| --- | --- | --- | --- | --- | --- | --- | --- |
| *ZmNPF4.10* | GRMZM2G137421 |  | *ZmNPF7.12* | GRMZM2G061303 |  | *ZmAMT1.1A* | GRMZM2G175140 |
| *ZmNPF6.2* | GRMZM2G064091 |  | *ZmNRT2.1* | GRMZM2G010280 |  | *ZmAMT1.1B* | GRMZM2G118950 |
| *ZmNPF6.3* | GRMZM2G476069 |  | *ZmNRT2.2* | GRMZM2G010251 |  | *ZmAMT1.3* | GRMZM2G028736 |
| *ZmNPF6.4* | GRMZM2G086496 |  | *ZmNRT2.3* | GRMZM2G163866 |  | *ZmAMT2.1* | GRMZM2G080045 |
| *ZmNPF6.5* | GRMZM2G161483 |  | *ZmNRT2.5* | GRMZM2G455124 |  | *ZmAMT3.1* | GRMZM2G335218 |
| *ZmNPF6.6* | GRMZM2G161459 |  | *ZmNRT3.1A* | GRMZM2G179294 |  | *ZmAMT3.2* | GRMZM2G338809 |
| *ZmNPF6.7* | GRMZM2G112154 |  | *ZmNRT3.1B* | GRMZM2G163494 |  | *ZmAMT3.3* | GRMZM2G043193 |
| *ZmNPF6.8* | GRMZM2G176253 |  | *ZmAMF1.1* | GRMZM2G062024 |  | *ZmAMT4* | GRMZM2G473697 |
| *ZmNPF7.10* | GRMZM2G044851 |  | *ZmAMF1.2* | GRMZM2G164743 |  |  |  |

*Table S2*

One-way ANOVA results of root gene expression between control (C), starved (S) and resupplied (R) plants. Bold text denotes p<0.05.


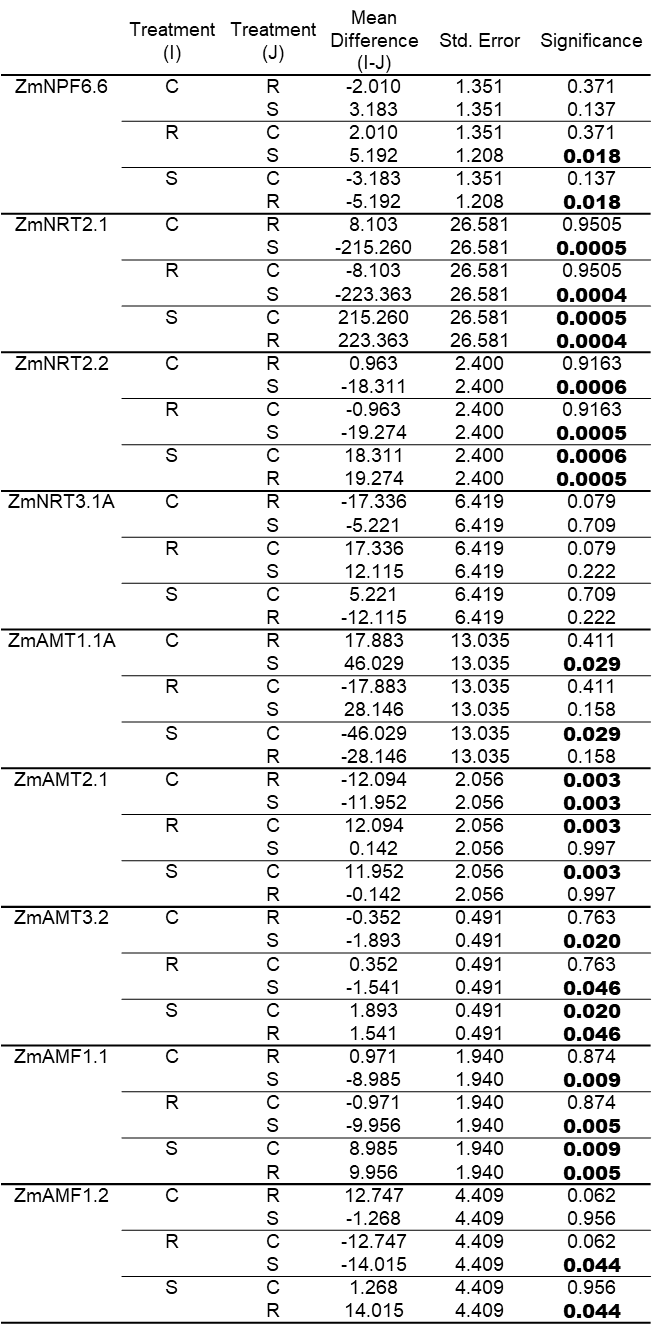


*Table S3*

One-way ANOVA results of shoot gene expression between control (C), starved (S) and resupplied (R) plants. Bold text denotes p<0.05.


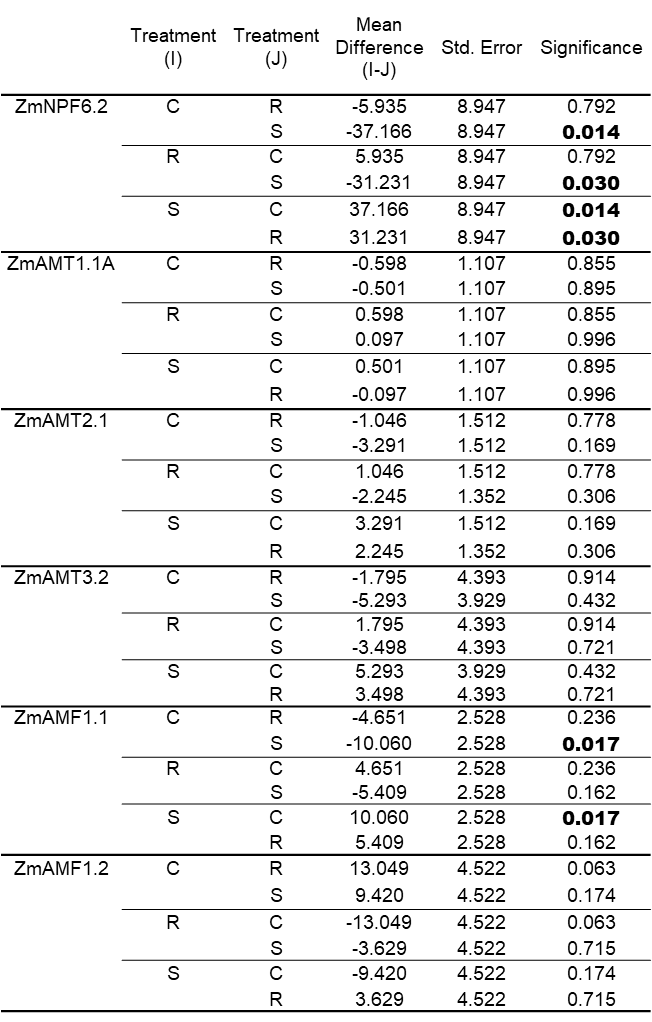

Supplement: Supplementary file 1 — Table S1. List of genes studied. Table S2. One-way ANOVA results of root gene expression between control (C), starved (S) and resupplied (R) plants. Bold text denotes p < 0.05. Table S3. One-way ANOVA results of shoot gene expression between control (C), starved (S) and resupplied (R) plants. Bold text denotes p < 0.05. (DOCX 118 kb) [file 12870_2019_1768_MOESM1_ESM.docx]
